# Parental satisfaction and acceptance of silver diamine fluoride treatment for molar incisor hypomineralisation in pediatric dentistry: a cross-sectional study

**DOI:** 10.1038/s41598-024-55456-0

**Published:** 2024-02-24

**Authors:** Zuhair Al-Nerabieah, Muaaz AlKhouli, Mayssoon Dashash

**Affiliations:** Pediatric Dentistry Department, Faculty of Dentistry, Damascus University, Damascus, Syria

**Keywords:** Parental satisfaction, Silver diamine fluoride, Molar incisor hypomineralisation, Pediatric dentistry, Epidemiology, Psychology and behaviour, Health care, Medical research

## Abstract

The study aimed to investigate parental satisfaction and acceptance of silver diamine fluoride (SDF) treatment for permanent molars affected by molar incisor hypomineralisation (MIH). This study was conducted in the pediatric dental department at Damascus University, Syria. This study was performed at the period from Jan 2023 to April 2023. In this cross-sectional study, a validated questionnaire employing a 5-point Likert scale was used to evaluate esthetics, ease of application, pain perception, and taste acceptability. Participants included 100 parents or guardians of children aged 6–9 years who had received SDF treatment in the past year. The findings of this study revealed high satisfaction levels (77.5%) with the treatment. Parents expressed contentment with the appearance of their child's molars after SDF application (58% agreed or strongly agreed) and found the application process easy and pain-free (100% agreed or strongly agreed). However, taste acceptability posed a challenge, with over half of the parents (53%) finding it unacceptable. Regression analysis underscored the significant impact of esthetics, ease of application, pain perception, and taste on parental satisfaction. Moreover, parents with higher education levels (graduate or postgraduate) exhibited higher acceptance rates compared to those with lower education levels (63.1% vs. 33.6%). Notably, parental gender and age did not significantly influence SDF treatment acceptance. This study provides critical insights into parental satisfaction and acceptance of SDF treatment for MIH-affected permanent molars. Despite concerns about tooth discoloration, the high satisfaction levels suggest that SDF holds promise as an effective treatment option. Parental education significantly influenced acceptance rates. This research highlights the importance of considering parental perspectives and tailoring communication strategies in pediatric dentistry, ultimately contributing to improved care for young patients with MIH.

## Introduction

In the realm of pediatric dentistry, molar incisor hypomineralisation (MIH) stands as a condition of increasing significance. This dental ailment predominantly affects the molars and incisors of young children, casting a shadow over their oral health^1,2^.

Worldwide, the prevalence of MIH varies, but it remains a global concern^3^. Notably, in Syria, MIH has exhibited a particularly high prevalence, with an alarming 39.9% of children affected^4^. This underscores the urgent need for effective and accessible treatment options.

MIH is often accompanied by enamel defects and a heightened susceptibility to dental caries^5,6^. Managing the multifaceted challenges presented by MIH-affected molars has become a focal point in pediatric dentistry, necessitating innovative and patient-friendly treatment approaches^7^.

One such innovation that has garnered considerable attention in the pediatric dentistry community is the utilization of silver diamine fluoride (SDF)^8,9^. This minimally invasive and cost-effective treatment has emerged as a beacon of hope for dental practitioners faced with the task of managing MIH^10^.

SDF exerts its therapeutic effects through a multifaceted mechanism of action, making it a potent tool in the management of dental caries, particularly in cases like MIH^11,12^.

Originating in Japan in the 1960s, SDF's chemical composition, encompassing 25% silver for antimicrobial action, 8% ammonia as a solvent, and 5% fluoride for remineralization, underlines its versatility and therapeutic potential. Also, FDA clearance in 2014 further attests to its safety and efficacy, marking it as a valuable tool in pediatric dentistry^13^.

Upon application, the silver component plays a crucial role by exhibiting strong antimicrobial properties. It inhibits the activity of bacteria, preventing further progression of dental caries. The high concentration of fluoride is a key feature as fluoride is well-established for its remineralizing effects on tooth enamel and dentin^14^.

When SDF is applied to affected teeth, it induces a chemical reaction with hydroxyapatite, a key component of tooth structure. This reaction results in the formation of silver phosphate and calcium fluoride. The silver ions released during this process actively inhibit mineral demineralization, providing a protective shield against the acid-producing bacteria responsible for caries. Simultaneously, the fluoride ions contribute to the remineralization of hydroxyapatite, enhancing the mineral density and hardness of the affected tooth structure^15^.

One of the notable aspects of SDF's mechanism is its ability to occlude dentinal tubules through the production of fluorohydroxyapatite and silver phosphate, thus increasing mineral density and hardness^16^. This is particularly significant in cases of hypersensitivity, a common issue in conditions like MIH. By blocking these tubules, SDF reduces the exposure of the tooth's inner nerves to external stimuli, providing profound and long-lasting relief from dentinal hypersensitivity. This aspect makes SDF not only effective in arresting caries but also invaluable in improving the overall comfort of patients undergoing treatment^17,18^.

SDF's unique properties make it a promising tool in the fight against dental caries, particularly in cases involving MIH-affected molars^11,12^. Its non-invasive nature and relative ease of application offer a gentle alternative to more invasive treatments, potentially alleviating the discomfort and anxiety often associated with dental procedures in young patients^10,19^.

The unique combination of silver and fluoride in SDF offers a powerful defence against the ravages of dental caries, making it a promising treatment option, particularly for MIH-affected molars^17,18^.

Parental acceptance of SDF application on their children's teeth is a subject of growing interest within the field of pediatric dentistry^13^. SDF's clinical efficacy is well-documented, but its acceptance as a treatment option hinges on how parents perceive its outcomes. A central concern among parents and dentists alike is the potential for tooth discoloration associated with SDF application. The perceived impact on the aesthetic appearance of a child's teeth can understandably raise apprehensions^20^.

In 2018, Clemens et al., created a questionnaire to incorporate four pivotal aspects related to parental perceptions of the SDF application process. These aspects include evaluating the ease of the SDF application, gauging comfort with the discoloration of cavities post-SDF placement, assessing the pain-free experience of SDF application for their child, and exploring the acceptability of the taste of SDF^21^. Notably, Clemens et al.^20^ questionnaire stands out as the only one in the literature to include children tolerability for SDF taste, thus contributing significantly to the understanding of parental experiences in the context of pediatric dental treatments.

This concern over discoloration places parental acceptance at the forefront of decision-making when pediatric dentists consider recommending SDF treatment for young patients, particularly those with MIH. Consequently, understanding parental acceptance becomes crucial as it serves as the primary barrier or bridge to the effective utilization of SDF in managing pediatric dental cases^22^.

While previous research has delved into the causes, symptoms, and various treatment modalities for MIH, a noticeable gap remains in the understanding of parental perspectives^23^. This gap takes center stage when we consider the experiences and insights of parents whose children have undergone SDF treatment for MIH. This parental perspective is not merely an academic curiosity. It is a critical factor that can significantly influence treatment outcomes, compliance, and the overall well-being of the child.

Notably, the existing body of literature lacks systematic studies that have holistically assessed parental satisfaction and acceptance of SDF treatment, particularly in the context of permanent molars affected by MIH. There is a need to fill this critical knowledge gap by providing a deep and nuanced understanding of the factors that influence parental acceptance of SDF treatment.

Therefore, this comprehensive cross-sectional investigation was undertaken to explore parental satisfaction and acceptance towards SDF treatment on permanent molars affected by MIH. Other factors such as potential tooth discoloration, as well as the comfort levels of both parents and their children with the treatment procedure were also explored.

## Methodology

### Study design

The present cross-sectional study assess parental satisfaction and acceptance of SDF treatment for molars affected by MIH. The study was conducted in accordance with the ethical standards outlined in the Declaration of Helsinki^24^. Ethical approval was obtained from the Institutional Review Board at Damascus university with the following number (NO. 2984), and a signed informed consent was obtained from all participants.

This study was conducted in the pediatric dental department at Damascus University, Syria. This study was performed at the period from Jan 2023 to April 2023.

The current study adheres to the STrengthening the Reporting of OBservational studies in Epidemiology (STROBE) guidelines for cross-sectional studies, ensuring transparency and robust reporting^25^.

### Study participants

Participants were recruited from the pediatric dental department at Damascus University. Eligible participants were specifically identified as parents or legal guardians of children aged 6–9 years, whose children had received a diagnosis of mild molar incisor hypomineralisation (MIH) in their permanent molars. This classification adhered to the criteria set forth by the European Academy of paediatric dentistry (EAPD)^12^. The inclusion criteria further emphasized that the identified molars exhibited induced sensitivity only without the presence of caries or post-eruptive enamel breakdown. Moreover, to qualify for participation, the affected molars must have undergone silver diamine fluoride (SDF) treatment within the past 12 months. Exclusion criteria included parents who did not speak the primary language of the study or had children with severe cognitive impairments.

### Sample size calculation

The sample size for this study was calculated using the formula: $n=\frac{{z}^{2}p(1-p)}{{d}^{2}}$, a widely recommended method for determining sample size in descriptive cross-sectional studies^26^. In adherence to a 5% type I error rate, the standard normal variate (Z) was set at 1.96. The proportion of the population was derived from previous studies, specifically the mean value of parental satisfaction regarding SDF application, which was found to be 60%. To ensure precision in our estimates, the desired margin of error (d) was set at 0.1. Consequently, the sample size calculation indicated a requirement for 92 participants. To account for potential attrition or unforeseen circumstances, we recruited 100 parents for the study, thereby enhancing the robustness and reliability of our findings. This methodology was chosen to guarantee that the study had adequate statistical power to detect meaningful relationships between parental satisfaction and various factors.

It should be noted that the sample population for this study had previously participated in a randomized controlled trial (RCT) comparing the preventive efficacy of SDF and MI Varnish on molars affected by MIH in children^9^. A total of 100 participants met the eligibility criteria and consented to participate in the study.

### Application protocol of SDF

The application of SDF in this study followed the University of California San Francisco (UCSF) Protocol^27^. The procedure commenced with a thorough cleaning of the affected teeth using gauze, creating a dry field that was maintained with the assistance of cotton rolls. A single drop of SDF (Advantage Arrest, USA) was dispensed into a disposable plastic dish. Subsequently, a microbrush applicator (MRG400, Henry Schein, USA) was employed to apply the SDF to the affected tooth. The application was conducted for a duration of one minute, ensuring thorough coverage.

To support the effectiveness of the SDF application, parents were provided with specific post-application instructions. Participants were advised to refrain from consuming food or hot beverages for a period of 1 h. This precautionary measure aimed to optimize the retention and efficacy of the applied SDF.

### Questionnaire development

For this study, a questionnaire was employed to assess parental satisfaction and acceptance of SDF treatment. The questionnaire was originally developed in English by Clemens et al.^21^, has since been validated and tested for reliability. This well-established questionnaire was selected for its comprehensive coverage of pertinent aspects.

### Translation and adaptation

To ensure linguistic and cultural relevance, the English version of the questionnaire was translated into Arabic. This translation process involved collaboration with two professionals whose native language was Arabic. Each professional independently translated the questionnaire into Arabic. Subsequently, the two Arabic versions were meticulously compared, and the most linguistically and culturally appropriate translation version was selected.

A critical step in the translation process involved back-translating the chosen Arabic version into English which was done by two professionals whose native language was English. This back-translation allowed for a thorough examination and comparison between the original English version and the back-translated Arabic version to ensure semantic equivalence and cultural appropriateness.

### Pre-testing and refinement

In the pre-testing stage, a convenient sample of 20 parents/guardians, selected based on the same criteria as in the main study (except that their data were not included in the study's results), were invited to participate. These individuals completed the questionnaire. During this phase, deviations and errors in translation were meticulously scrutinized, and any necessary adjustments were made to improve linguistic clarity and ensure that the target participants easily understood questions.

### Testing the translated version of the questionnaire

#### Content validity

Measuring content validity involves assessing items of a questionnaire by asking experts if each item reaches the main aim that, the questionnaire is designed to cover. Five experts (three professors in pediatric dentistry and two professors in psychology) were asked to assess the items. Lawshe formula was used to determine the content validity ratio (CVR)^28^. Therefore, we asked each expert to determine whether the information behind each item is "essential", "useful but not necessary" or "not necessary".

$$CVR = \frac{{N_{e} - \left( \frac{N}{2} \right)}}{N/2}$$

N_e_ is the number of essentials for the item, N is the number of experts.

The content validity index CVI, which is the mean of CVR for all studied items, was 0.92 and this means that the questionnaire developed was with high content validity.

### Test–retest reliability

It involves administering the same questionnaire to 20% of the sample under the same conditions after period of time. In this study, 20% of the total sample (20 Participants) were randomly selected to refill up the questionnaire once again after 10 days.

Test-rest reliability was estimated with correlations between the scores at time point 1 and those at time point 2. Correlation coefficient (r) of parental satisfaction scores between the two time points was 0.89. This means that the studied questionnaire has an excellent reliability.

### Internal consistency reliability

Internal consistency concerns the extent to which items on the test or instrument are measuring the same thing. This can be measured by Cronbach's alpha statistics. Cronbach's alpha reliability coefficient was 0.95, which means that the studied questionnaire has an excellent internal consistency.

### Data collection

In this study, parental satisfaction with SDF treatment for their children's molars affected by MIH was assessed using a 4-item questionnaire. The questionnaire employed a 5-point Likert scale, with responses ranging from "1: Strongly Disagree" to "5: Strongly Agree."

Structured interviews were conducted with parents or guardians to gather data regarding their satisfaction and acceptance of SDF treatment. The questionnaire included the following Likert scale questions:

1. Esthetics: "You are comfortable with your child's esthetics after SDF placement."
2. Ease of Application: "SDF application is an easy process."
3. Pain Perception: "SDF application is pain-free for your child."
4. Taste: "SDF taste is acceptable to your child."

Each of these questions was answered on a 5-level Likert scale: Strongly Agree, Agree, Neutral, Disagree, and Strongly Disagree.

### Statistical analysis

The statistical analysis for this study was conducted using the Statistical Package for the Social Sciences (SPSS). Descriptive statistics were utilized to summarize the demographic characteristics of the 100 participants, including age, gender, and education levels.

Chi-square tests were employed to analyze the association between parental education levels and the acceptance of silver diamine fluoride (SDF) treatment.

Regression analysis was performed to assess the impact of independent variables (Esthetics, Ease of Application, Pain Perception, and Taste) on the dependent variable, Parental Satisfaction. Beta coefficients were calculated to determine the strength and direction of these relationships, while *P*-values were used to establish the statistical significance of the relationships.

### Ethics approval and consent to participate

The study was conducted in accordance with the ethical standards outlined in the Declaration of Helsinki. Ethical approval was obtained from the Institutional Review Board at Damascus university with the following number (NO. 2984), and a signed informed consent was obtained from all participants.

## Results

A total of one hundred parents participated in this study. The majority were mothers (n = 69), and aged between 31 and 40 years (n = 75). In addition, 42% of the participants were graduates, while 13% were post-graduates (Table 1).

**Table 1:** Demographic characteristics of study participants.

Characteristics	n
Age of child in years (mean ± SD)	7.6 ± 1.4
Gender of child	
Girls	42
Boys	58
Age group of parents (years)	
20–30	5
31–40	75
41–50	17
Older than 50	3
Gender of parents	
Mothers	69
Fathers	31
Level of education	
Illiterate	2
Primary	10
High school	33
Graduate	42
Postgraduate	13
Socioeconomic status	
Low	100

Regarding parental satisfaction with the appearance of their child's molars after SDF application, the findings revealed that 7% of parents strongly disagreed, 20% disagreed, 15% were neutral, 30% agreed, and 28% strongly agreed with the results.

When assessing the ease of application, 100% of parents either strongly agreed (70%) or agreed (30%) that the procedure was easy. On the other hand, regarding pain perception during SDF application, all parents found it to be a pain-free procedure, with 65% strongly agreeing and 35% agreeing.

As for the acceptability of the SDF taste by the children, the majority of parents reported that their children did not find the taste acceptable, with 18% strongly disagreeing, 35% disagreeing, 20% being neutral, 20% agreeing, and 7% strongly agreeing.

Table 2 presents the percentage distribution of each point on the scale for each questionnaire item, along with the mean values for all responses to each item. The highest mean satisfaction value was observed for ease of application (4.7), followed by pain perception (4.65), and then esthetics, with a mean value of (3.52). The lowest mean satisfaction value was associated with taste acceptability (2.63).

**Table 2:** Questionnaire responses and mean satisfaction scores of SDF treatment for MIH-affected permanent molars.

Domain	Question	Strongly disagree	Disagree	Neutral	Agree	Strongly agree	Mean of values	SD
Esthetics	‘You are comfortable with your child's esthetics after SDF placement’	7	20	15	30	28	3.52	± 0.9
Ease of application	‘SDF application is an easy process’	0	0	0	30	70	4.70	± 0.2
Pain perception	‘SDF application is pain-free for your child’	0	0	0	35	65	4.65	± 0.3
Taste	‘SDF taste is acceptable to your child’	18	35	20	20	7	2.63	± 0.8

The mean value of parental satisfaction regarding SDF application for all studied items was 3.875, which corresponds to 77.5%, indicating high satisfaction.

In the regression analysis, the impact of four independent variables (Esthetics, Ease of Application, Pain Perception, and Taste) on the dependent variable, Parental Satisfaction, was assessed.

The results demonstrated that all four independent variables had a statistically significant impact on Parental Satisfaction. With an Adjusted R-squared value of 0.632, these variables collectively explained a substantial portion of the variance in Parental Satisfaction, emphasizing their significance. Findings are presented in Table 3.

**Table 3:** Regression analysis of independent variables on parental satisfaction with SDF treatment for MIH-affected permanent molars.

Independent variable	Beta Coefficient	*P*-value
Esthetics	0.356	0.000*
Ease of application	0.275	0.000*
Pain perception	0.143	0.0004*
Taste	0.210	0.0002*
Dependent variable	Adjusted R-squared	
Parental satisfaction	0.632	

*Statistically significant (*P*-value < 0.001).

Moreover, a detailed analysis of the satisfaction of SDF treatment revealed that the Chi-square test showed that higher number of parents with higher education levels (graduate and postgraduate) accepted SDF treatment compared to lower education level groups significantly (Illiterate, primary school and high school) (63.1% vs. 33.6%, respectively, *P* < 0.005). However, parental gender and age had no significant association with acceptance of SDF treatment (Table 4).

**Table 4:** Association between parental satisfaction with SDF treatment and various independent variables, including age, sex, and education level.

	Percentage of parents satisfaction	Test value	*P*-value
Gender		X^2^-value	
Fathers	72.5	9.04	0.1
Mothers	67.2		
Age		F-ratio	
20–30 years	70.4	22.18	0.2
31–40 years	63.2		
41–50 years	65.8		
Older than 50 years	73.1		
Education		X^2^-value	
Low level	33.6	1.44	0.001*
High level	63.1		

*Statistically significant (*P*-value < 0.05).

## Discussion

The present study employed a cross-sectional research design to assess parental satisfaction and acceptance of SDF treatment for permanent molars affected by MIH. This study adhered to STROBE guidelines for cross-sectional studies, ensuring a structured and rigorous approach to data collection and analysis.

The primary aim of this study was to explore parental acceptance and satisfaction regarding SDF treatment for MIH-affected molars, a topic that had not been comprehensively addressed in previous literature. To achieve this aim, a Likert scale questionnaire was selected as the most suitable instrument to gauge parental opinions and satisfaction levels.

The questionnaire, which was adapted from Clemens et al.^21^, underwent a rigorous translation process to ensure its applicability to Arabic-speaking participants. The pre-testing phase, involving a sample of 20 parents/guardians, helped identify and rectify any discrepancies in translation.

Regarding parental satisfaction, our findings indicate that a significant proportion of parents expressed satisfaction with the aesthetic outcomes of SDF treatment on their children's permanent molars. This aspect is crucial, as it addresses one of the primary concerns among parents—the potential for tooth discoloration following SDF application. The study's results suggest that, despite concerns about discoloration, the majority of parents remained satisfied with the aesthetic results of SDF treatment (77.5%).

Comparing the findings of this study with prior research investigating parental acceptance of SDF treatment on primary teeth, certain consistencies and distinctions emerge. Prior studies have highlighted concerns about the aesthetic implications of SDF application^22,29^, an issue that was confirmed in our study. Parents exhibited a statistically notable inclination towards accepting SDF treatment when administered to posterior teeth^30^. This trend is consistent with observations found in various studies involving other types of unesthetic restorations, including stainless-steel crowns. It is a well-established phenomenon that patients generally express a higher preference for aesthetic interventions, especially when the restoration is prominently visible^31^.

It is noteworthy that, to our knowledge, no prior research has systematically assessed parental acceptance of SDF treatment specifically on permanent molars affected by MIH. This study thus fills a significant gap in the literature by providing valuable insights into the factors influencing parental acceptance in this context. It is crucial to consider that MIH-affected molars often pose a more intricate set of challenges than primary teeth, further underscoring the importance of this investigation.

Furthermore, the results of this study provide valuable insights into the factors influencing parental satisfaction and acceptance of SDF treatment for molars affected by MIH. Two key findings deserve particular attention and can be compared to existing research in the field.

Firstly, one of the standout findings in our study was that all parents reported that their children found the SDF application to be pain-free. This result is highly encouraging, emphasizing the non-invasive and gentle nature of SDF treatment. The fact that 65% of parents strongly agreed with this statement, and an additional 35% agreed, underscores the consensus regarding the painless nature of the procedure.

These findings closely align with previous research that has praised SDF for its minimal discomfort during application^30,32^. The pain-free aspect of SDF treatment is particularly beneficial in pediatric dentistry, where reducing anxiety and apprehension in young patients is a top priority. It also enhances the overall experience of dental care, potentially reducing dental anxiety in children and fostering positive attitudes toward oral health^33^.

Secondly, the link between the perceived ease of application of SDF and higher levels of parental satisfaction and acceptance aligns with broader principles of user experience and satisfaction. In healthcare, treatments perceived as less burdensome or invasive often lead to higher patient satisfaction and adherence rates^34^.

In the case of pediatric dentistry, where children's comfort and cooperation during treatment are paramount, a minimally invasive and straightforward application process can significantly influence parental decisions^35^. This result is consistent with the literature on healthcare acceptance, which highlights the importance of user-friendly procedures in promoting treatment success^30,36^.

Dental practitioners may find value in emphasizing the ease and convenience of SDF treatment when discussing it with parents, as this aspect appears to play a pivotal role in their acceptance^37,38^. Furthermore, the results of this study showed that taste acceptability emerged as a notable factor influencing parental satisfaction and acceptance of SDF treatment for MIH-affected molars.

The findings revealed a considerable divide in parental perceptions, with a majority reporting that their children did not find the taste of SDF treatment acceptable. This discrepancy in taste acceptability raises intriguing questions about the role of sensory experiences in treatment acceptance, especially among pediatric patients. While SDF's clinical efficacy is well-documented, the taste factor appears to be a noteworthy consideration, potentially influencing parental decisions and children's cooperation during treatment.

It should be emphasised that the acceptability of taste is subjective and can vary widely among individuals and cultural contexts. A recent systematic review showed that some studies have reported that children, in particular, may exhibit a range of responses to different tastes, influenced by factors such as age, previous experiences, and cultural backgrounds^20^. Therefore, while taste acceptability emerged as a notable concern in our study, it's essential to view this finding within the broader landscape of pediatric dental care.

One important aspect to consider is the potential trade-off between taste and treatment effectiveness. SDF has gained recognition for its remarkable ability to halt dental caries progression, and its taste, while a concern for some, is arguably a small price to pay for its non-invasiveness and effectiveness^21^. Previous studies have indicated that parents and children may prioritize treatment outcomes over taste-related discomfort^20^. Therefore, while taste concerns exist, they may not outweigh the clinical benefits of SDF treatment.

Another notable discovery that merits specific consideration and can be likened to previous research in this domain is the educational background of parents. The observation that parents with higher education levels tend to express greater satisfaction and acceptance of the treatment aligns with broader trends in healthcare decision-making^39^. Prior studies have often reported that individuals with higher educational backgrounds tend to seek out and engage more actively in healthcare information and decision processes. They are often more receptive to novel treatments and technologies due to their ability to access and comprehend relevant information^40,41^.

In the context of this study, parents with higher education levels may have had a better understanding of the benefits and limitations of SDF treatment, leading to increased satisfaction and acceptance. This finding was similar to previous research^42^, which underscores the importance of tailored educational strategies that consider the educational backgrounds of parents, particularly when introducing innovative treatments like SDF.

While this study contributes significantly to the understanding of parental satisfaction with SDF treatment for MIH-affected molars, certain limitations must be acknowledged. The cross-sectional design restricts our ability to establish causal relationships, warranting caution when interpreting the results. Additionally, the study's recruitment of participants from a specific geographic area may limit the generalizability of the findings. Furthermore, reliance on self-reported data introduces the possibility of response bias.

## Conclusion

In conclusion, this study offers unique insights into parental satisfaction and acceptance of SDF treatment for MIH-affected permanent molars. The remarkably high satisfaction levels suggest that SDF holds promise as an effective treatment option, despite concerns about tooth discoloration. The influence of parental education on acceptance rates emphasizes the need for tailored communication strategies in dental practice. This research contributes to the growing body of knowledge in pediatric dentistry and underscores the importance of considering parental perspectives when formulating treatment strategies.

## Data Availability

The datasets used and/or analysed during the current study are available from the corresponding author on reasonable request.

## Abbreviations

SDF: Silver diamine fluoride
MIH: Molar incisor hypomineralisation
STROBE: STrengthening the Reporting of OBservational studies in Epidemiology
EAPD: European Academy of paediatric dentistry
